# Rational and combinatorial tailoring of bioactive cyclic dipeptides

**DOI:** 10.3389/fmicb.2015.00785

**Published:** 2015-07-30

**Authors:** Tobias W. Giessen, Mohamed A. Marahiel

**Affiliations:** ^1^Department of Systems Biology, Harvard Medical School, BostonMA, USA; ^2^Wyss Institute for Biologically Inspired Engineering, Harvard University, BostonMA, USA; ^3^Department of Chemistry, Philipps-University MarburgMarburg, Germany; ^4^LOEWE Center for Synthetic Microbiology, Philipps-University MarburgMarburg, Germany

**Keywords:** diketopiperazines, cyclic dipeptides, combinatorial biosynthesis, biocatalysis, tailoring enzymes, synthetic biology

## Abstract

Modified cyclic dipeptides represent a diverse family of microbial secondary metabolites. They display a broad variety of biological and pharmacological activities and have long been recognized as privileged structures with the ability to bind to a wide range of receptors. This is due to their conformationally constrained 2, 5-diketopiperazine (DKP) scaffold and the diverse set of DKP tailoring enzymes present in nature. After initial DKP assembly through different biosynthetic systems modifying enzymes are responsible for installing functional groups crucial for the biological activities of the resulting modified DKPs. They represent a vast and largely untapped enzyme repository very useful for synthetic biology approaches aiming at introducing structural variations into DKP scaffolds. In this review we focus on these DKP modification enzymes found in various microbial secondary metabolite gene clusters. We will give a brief overview of their distribution and highlight a select number of characterized DKP tailoring enzymes before turning to their application potential in combinatorial biosynthesis with the aim of producing molecules with improved or entirely new biological and medicinally relevant properties.

## Introduction

Peptide natural products are one of the most fruitful sources of medicinally relevant compounds (Borthwick, 2012). Cyclic peptides in particular show superior bioactivities compared with their linear analogs due to their enhanced stability, protease resistance and conformational rigidity, all factors that improve their ability to specifically interact with biological targets (Liskamp et al., 2011; Menegatti et al., 2013). The smallest possible cyclic peptides are the cyclic dipeptides (CDPs) containing a 2,5-diketopiperazine (DKP) heterocycle resulting from the double condensation of two α-amino acids. DKP-containing natural products represent a large class of bioactive compounds produced by bacteria, fungi, plants, and animals. Those privileged structures, selected over evolutionary time for bioactivity and stability, possess characteristics that make them attractive scaffolds for drug discovery. Besides the properties shared with other cyclic peptides, their rigid backbone gives CDPs the ability to mimic preferential peptide conformations and allows them to contain highly constrained amino acids while at the same time being flexible enough to exist either in an essentially planar or lightly puckered boat form (Ciarkowski, 1984). Their three-dimensionality, two hydrogen-bond donor and acceptor sites and the possibility to introduce various substituents to the DKP-ring and the respective side chains of the constituent amino acids gives them a marked advantage over many typically planar small molecules discovered through conventional combinatorial chemistry approaches. A number of DKP-based compounds identified by targeted screens of synthetic DKP libraries have been developed into drugs acting as PDE5 inhibitors (Tadalafil, treatment of pulmonary arterial hypertension, and erectile dysfunction, Daugan et al., 2003), oxytocin antagonists (Retosiban and Epelsiban, treatment of preterm labor; Liddle et al., 2008; Borthwick et al., 2012) and CCR5 entry inhibitors (Aplaviroc, treatment of HIV infection; Maeda et al., 2004). In addition, various compounds based on naturally occurring CDPs are currently being investigated as potential anticancer drugs [e.g., phenylahistin (*Aspergillus ustus*), dehydrophenylahistin, and NPI-2358 (Plinabulin) acting as tubulin depolymerizing agents (Kanoh et al., 1999; Kanzaki et al., 2002)], antibiotics [e.g., avrainvillamide (*Aspergillus* sp.), active against multidrug-resistant bacteria (Sugie et al., 2001), and bicyclomycin (*Streptomyces sapporonesis*), broad-spectrum antibiotic active against Gram-negative bacteria (Miyoshi et al., 1972)] and anti-inflammatory agents [e.g., FR106969 (*Penicillium citrinum*) and FR900452 (*Streptomyces phaeofaciens*), inhibitors of PAF-induced platelet aggregation (Shimazaki et al., 1991)].

Despite the fact that numerous CDP natural products have been isolated and characterized for use in pharmacology, relatively little is known about their functions in the producing organisms. Their suggested roles range from agents used for microbial warfare to their involvement in biochemical communication phenomena like quorum-sensing as well as interspecies and interkingdom signaling (Holden et al., 1999; Degrassi et al., 2002; Ortiz-Castro et al., 2011; Olson et al., 2014).

In addition to applications in medicine and pharmacology, DKP-containing compounds have also been used as catalysts and chiral auxiliaries in synthetic organic chemistry (Borthwick, 2012).

The DKP-scaffold can be accessed either by purely chemical means using different solid-phase or in-solution methodologies (Borthwick, 2012; Gonzalez et al., 2012) or by employing biosynthetic enzymes called non-ribosomal peptide synthetases (NRPSs) and cyclodipeptide synthases (CDPSs; Belin et al., 2012; Giessen and Marahiel, 2014). NRPSs and CDPSs are usually part of a dedicated biosynthetic gene cluster responsible for the assembly of modified CDPs. They very often encode one or more enzymes that introduce specific modifications to the DKP-scaffold crucial for the bioactivity or stability of the resulting natural product (Giessen et al., 2013a). Both chemical synthesis and enzyme-catalyzed assembly are valid ways of providing suitable substrates for DKP tailoring enzymes. When using chemically synthesized substrates, DKP modification enzymes can be employed in chemoenzymatic and cell-free *in vitro* settings as well as in feeding experiments while whole-cell *in vivo* biosynthesis based on *in situ* substrate generation by NRPS or CDPS enzymes represents an alternative approach to obtain modified CDPs.

With the advent and rapid development of whole genome sequencing and metagenomics in the last decade it became evident that there is a vast and largely untapped source of orphan and cryptic biosynthetic gene clusters putatively encoding DKP tailoring enzymes that may be of great value for medicinal chemists and synthetic biologists alike (Kwon et al., 2012; Schofield and Sherman, 2013).

In this review, we will first survey the distribution of characterized DKP modifying enzymes in different microbial biosynthetic gene clusters comparing their genetic contexts and their roles in various biosynthetic routes. We will highlight the characteristics of chemical transformations catalyzed by a selection of characterized enzymes. Finally, we will turn to the application potential of DKP modification enzymes for *in vivo* and *in vitro* combinatorial biosynthesis.

## DKP Modification Enzymes

### Distribution and Diversity

The majority of identified DKP-containing natural products have been isolated from marine and terrestrial fungi with *Aspergillus* and *Penicillium* species being particularly fruitful sources of new CDPs (Borthwick, 2012). A substantial number of modified DKPs has also been isolated from the bacterial phyla Actinobacteria, Proteobacteria, and Firmicutes while so far, only one archaeon (*Haloterrigena hispanica*) has been shown to produce DKPs (Belin et al., 2012; Tommonaro et al., 2012; Giessen and Marahiel, 2014). However, the fact that the availability of sequenced genomes is heavily skewed toward plant, animal, and human pathogens and that isolation of natural products focusses on a select number of microbial genera and ecological niches, means that the ability to produce DKP-containing compounds could be more widespread than previously suspected. Besides microbes, plants have also been shown to produce a range of DKP-containing alkaloids (Prasad, 1995) and one functional CDPS gene has been characterized in the animal (sea anemone) *Nematostella vectensis* (Seguin et al., 2011). In addition, non-enzymatic processes can lead to the formation of functional CDPs in various organisms including mammals where for example cyclo(L-His-L-Pro) is found throughout the central nervous system and plays a role in various regulatory processes (Minelli et al., 2008).

Enzymes that specifically modify DKP-containing natural products are usually associated with biosynthetic enzymes able to assemble the DKP-scaffold. In microbes the genes responsible for the production of a specific secondary metabolite are most often found in close proximity to one another in dedicated biosynthetic gene clusters reflecting their evolutionary history through horizontal transmission (Fischbach et al., 2008). To date, two unrelated biosynthetic routes are known able to assemble CDPs. NRPSs, large multidomain enzyme complexes (Koglin and Walsh, 2009; Strieker et al., 2010), have long been known as a source of many structurally complex DKP-containing natural products while only relatively recently, a second enzyme class able to generate DKPs has been identified, namely the tRNA-dependent CDPSs (Belin et al., 2012; Giessen and Marahiel, 2014). In the case of NRPSs, many dedicated pathways that assemble modified DKP-scaffolds are known to be responsible for the synthesis of fungal and bacterial siderophores as well as bacterial and fungal antibiotics and toxins (Belin et al., 2012). In addition, the premature release of dipeptidyl intermediates during chain elongation can result in CDP side products during NRPS biosynthesis (Stachelhaus et al., 1998; Schultz et al., 2008). In contrast, CDPS-dependent pathways for CDP formation are almost exclusively confined to bacteria with only a handful of putative CDPS pathways identified by computational homology searches in eukaryotic organisms (Seguin et al., 2011; Giessen and Marahiel, 2014). Modified cyclic peptides dependent on CDPSs include the antibiotic albonoursin (*Streptomyces noursei*; Lautru et al., 2002; Gondry et al., 2009), the siderochrome pulcherrimininic acid (*Bacillus* spp.; Cryle et al., 2010; Bonnefond et al., 2011) and the nocazine family (*Nocardipsis* spp.) of antibiotics (Giessen et al., 2013a; Zhang et al., 2013).

Putative tailoring enzymes that modify the initially assembled CDP scaffold can be found in almost all NRPS and CDPS gene clusters coding for a DKP-containing compound. Regarding CDPS-dependent pathways, a large variety of different putative enzyme classes can be found in close association with the respective CDPS gene (Belin et al., 2012; Giessen and Marahiel, 2014). They include different types of oxidoreductases, hydrolases, transferases, and ligases. The most prevalent putative tailoring enzymes in CDPS clusters are various kinds of oxidases including at least seven distinct types of P450s, five different types of α-ketoglutarate/Fe^II^-dependent oxygenases and three distinct flavin-containing monooxygenases. In addition to oxidoreductases, a large number of different *C*-, *N*-, and *O*-methyltransferases, α/β-hydrolases, peptide ligases, and acyl-CoA transferases have been found in CDPS gene clusters. Turning to NRPS-dependent pathways, a similar variety of modification enzymes has been reported. Again, enzymes that modulate the oxidation level of the DKP scaffold and side chains are the most numerous enzyme variety (Belin et al., 2012). One distinguishing feature of fungal NRPS gene clusters is the prevalence of different prenyltransferases that carry out prenylations and reverse prenylations at various positions of the assembled CDP scaffold (Yu et al., 2012). Judging by the diverse set of putative modification enzymes found within NRPS and CDPS gene clusters it is safe to assume that highly modified CDPs rather represent the norm and not the exception among DKP-containing natural products, possibly reflecting their varied functions in the producing organisms.

### Transformations Catalyzed by DKP Modifying Enzymes

**FIGURE 1:**
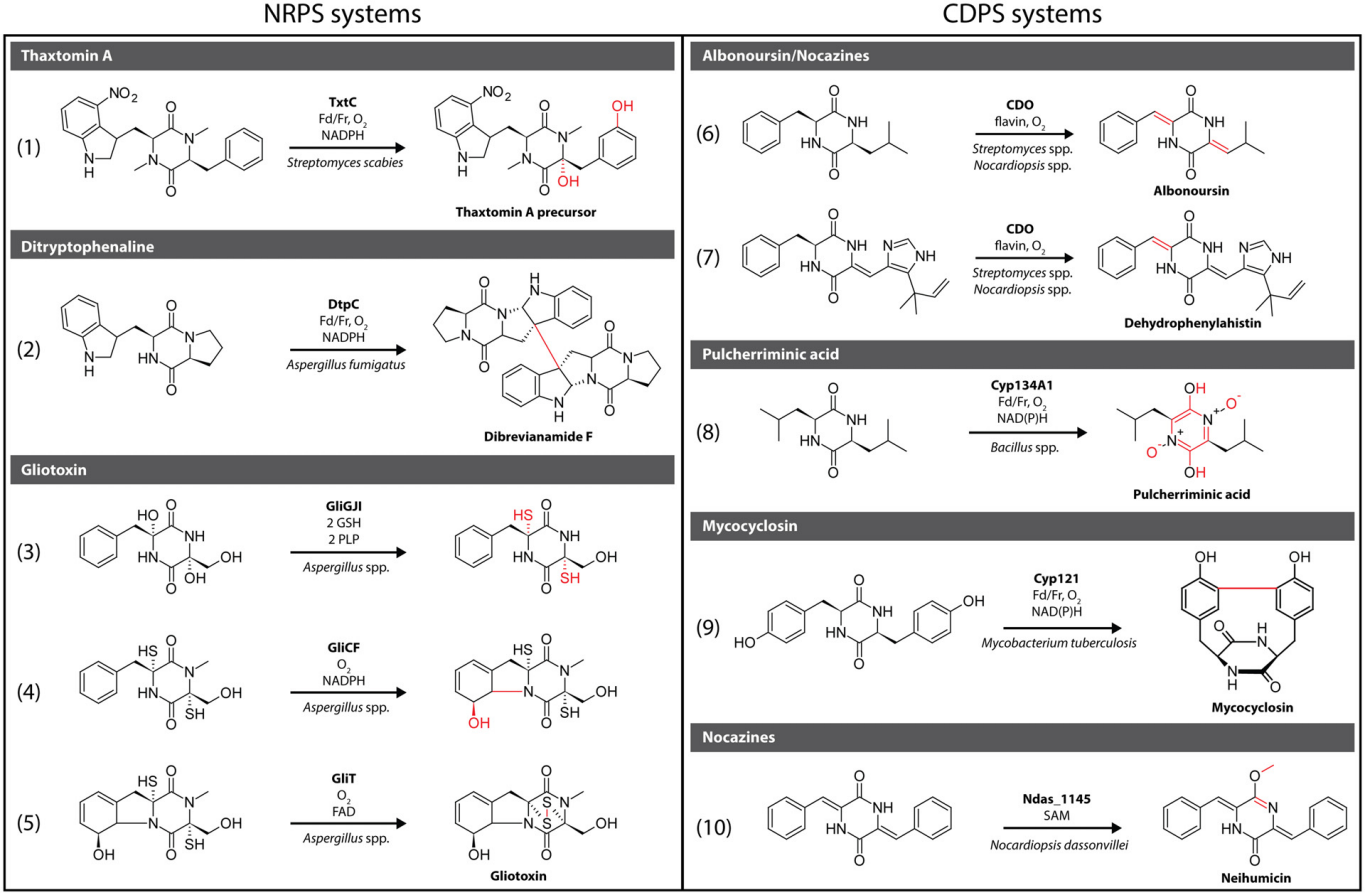
**Selection of characterized 5-diketopiperazine (DKP) tailoring enzymes originating from non-ribosomal peptide synthetase (NRPS) and cyclic dipeptide (CDPS) pathways.** Structural changes that are introduced by a given modification enzyme are highlighted in red.

Generally, CDPs can be modified at the DKP heterocycle or the side chains of the constituent amino acids. Modifications that connect the side chains with the DKP core through various cyclization strategies have also been frequently observed. In the following paragraphs, we will highlight a number of characterized DKP tailoring enzymes from both NRPS and CDPS biosynthetic pathways (**Figure 1**).

The cytochrome P450 TxtC is involved in the synthesis of the phytotoxin thaxtomin A (Healy et al., 2002; Barry et al., 2012) produced by different plant-pathogenic bacteria of the genus *Streptomyces*, including most notably *S. scabies*, the causative agent of the potato disease common scab (King and Calhoun, 2009). Its phytotoxicity is caused by inhibition of cellulose biosynthesis leading to plant cell necrosis (Scheible et al., 2003). TxtC introduces two hydroxyl groups regio- and stereoselectively at two distinct positions in a modified cyclo(L-Phe-L-Trp) scaffold [**Figure 1**, reaction (1)]. One hydroxylation takes place at the C_α_ of the Phe residue, while the second one modifies the *meta* position of its aromatic ring. C_α_ hydroxylation in particular has been shown to be essential for phytotoxicity with glycosylation or alkylation of the C_α_ hydroxyl leading to a loss of activity (Molesworth et al., 2010).

Dimeric DKP-containing natural products have been isolated from different *Aspergillus* species, including ditryptophenaline from *A. flavus* (Barrow and Sedlock, 1994). This compound inhibits substance *P* receptor and shows promising analgesic and anti-inflammatory activity (Popp et al., 1994; Berube, 2006). The cytochrome P450 DtpC involved in ditryptophenaline biosynthesis has been shown to be responsible for both pyrroloindole ring formation, linking the tryptophan side chain with the DKP core, and concurrent dimerization leading to a homodimeric product (Saruwatari et al., 2014). In addition, DtpC shows relaxed substrate specificity and has been used for the dimerization of the non-native monomeric precursor brevianamide *F* [**Figure 1**, reaction (2)]. A radical-mediated dimerization mechanism, initiated by hydrogen atom abstraction through the P450 heme moiety, has been proposed as the most likely reaction pathway (Saruwatari et al., 2014).

The epidithiodioxopiperazine (ETP) family of highly modified CDPs is produced by several fungal genera, including *Aspergillus, Eurotium*, and *Gliocladium* (Scharf et al., 2012). Their defining feature is a disulfide bridge spanning the DKP heterocycle that is responsible for their high cytotoxicity by facilitating the reaction with and thus inactivation of thiol containing proteins (Mullbacher et al., 1986). In addition, reactive oxygen species are generated by redox cycling contributing to the toxicity of members of the ETP family. Its most prominent member is the lipid-soluble gliotoxin which was shown to induce apoptosis, inhibit angiogenesis and prevent NF-κB activation by inhibiting the proteasome among other bioactivities (Kroll et al., 1999; Pardo et al., 2006; Ben-Ami et al., 2009). High levels of gliotoxin are produced by *Aspergillus fumigatus* and it has been suggested that gliotoxin might be a virulence factor associated with invasive aspergillosis (Hof and Kupfahl, 2009). A number of highly unusual transformations take place during gliotoxin biosynthesis including a stereospecific glutathione (GSH)- and pyridoxal phosphate (PLP)-dependent sulfurization of both C_α_ atoms in the DKP-scaffold catalyzed by the sequential action of the glutathione *S*-transferase GliG, the peptidase GliJ and the thioesterase GliI [**Figure 1**, reaction (3)] (Davis et al., 2011). After initial double C_α_ hydroxylation by the P450 GliC, two molecules of water are eliminated generating imine intermediates that are then attacked by the nucleophilic cysteine thiols of two GSH molecules catalyzed by GliG. Subsequent removal of the two GSH glutamate residues by the dipeptidase GliJ makes the alpha-amino groups of the two cysteine residues accessible. This allows their condensation with GliI-bound PLP followed by PLP-mediated α,β-elimination reactions resulting in the double C_α_ sulfurization of the DKP-scaffold. A second unusual transformation during gliotoxin biosynthesis is the concomitant *meta*-hydroxylation of the phenylalanine side chain and ring closure between the Phe side chain and its amino group [**Figure 1**, reaction (4)]. This reaction is catalyzed by either GliC or GliF, both P450s, and proceeds via epoxidation of the aromatic ring in the Phe side chain followed by epoxide opening through a nucleophilic attack by the Phe amino group (Scharf et al., 2012). Finally, the disulfide bridge, essential for gliotoxin’s bioactivity, is generated by the unusual FAD-dependent oxidoreductase GliT [**Figure 1**, reaction (5)] (Scharf et al., 2012).

One of the most useful and widely used DKP tailoring enzymes is cyclic dipeptide oxidase (CDO). This unusual flavin-dependent α,β-dehydrogenase has been identified in different actinobacterial CDPS gene clusters, including the clusters encoding the albonoursin (*S. noursei*) and nocazine (*N. dassonvillei*) assembly pathways [**Figure 1**, reaction (6)] (Lautru et al., 2002; Giessen et al., 2013a), and shows very relaxed substrate specificity (Gondry et al., 2001). CDO is composed of two distinct small subunits that assemble into an apparent megadalton protein complex. Depending on the substrate, CDO is able to sequentially carry out one or two dehydrogenation reactions. The exact reaction mechanism has not been elucidated, although three different scenarios have been proposed, namely, direct dehydrogenation, α-hydroxylation followed by loss of water, and imine formation with subsequent rearrangement to the enamine (Gondry et al., 2001). The most intriguing application of CDO’s relaxed substrate specificity was the use of a CDO-containing cell-free extract from the albonoursin producer *S. albulus* KO-23 as a specific α,β-dehydrogenation catalyst transforming the fungal metabolite phenylahistin into dehydrophenylahistin [**Figure 1**, reaction (7)] (Kanzaki et al., 2002). This simple transformation resulted in a 2000 times higher activity of dehydrophenylahistin as a cell cycle inhibitor compared with phenylahistin. Dehydrophenylahistin was claimed to be highly cytotoxic, even more so than the known anticancer drugs taxol, vincristine, and vinblastine. Further screening resulted in the identification of the doubly dehydrogenated phenylahistin derivative NPI-2358 that has undergone successful phase I and phase II clinical trials for non-small cell lung cancer (Nicholson et al., 2006).

Pulcherriminic acid, produced by different bacterial and yeast species, is a precursor for the red extracellular pigment pulcherrimin formed in the presence of high levels of Fe^III^ in the growth medium (Cryle et al., 2010; Bonnefond et al., 2011). Pulcherriminic acid is able to chelate Fe^III^ with its two hydroxamic acid moieties and has been suggested to act either as an antibiotic, similar to aspergillic acid, or as a siderophore (Neilands, 1967). The highly unusual reaction that transforms cyclo(L-Leu-L-Leu) into pulcherriminic acid in *Bacillus* species is catalyzed by the P450 Cyp134A1 (CypX). The oxidation of the DKP-scaffold of various CDP substrates by Cyp134A1 involves three oxidative steps leading to double *N*-oxide formation with concomitant aromatization of the DKP-ring system [**Figure 1**, reaction (8)]. The reaction mechanism has been proposed to proceed either via hydroxylation and elimination of water or a direct electron transfer reaction. In addition, Cyp134A1 has been shown to be able to oxidize a number of steroidal substrates (Furuya et al., 2008, 2009).

The secondary metabolite mycocyclosin produced by *Mycobacterium tuberculosis*, the causative agent of tuberculosis, possesses a highly unusual three dimensional structure where the two *meta* positions of the tyrosine side chains of cyclo(L-Tyr-L-Tyr) are connected via a carbon-carbon single bond [**Figure 1**, reaction (9)] (Gondry et al., 2001, 2009). The two *meta* carbons of the DKP substrate are linked through the action of the P450 Cyp121, which has been shown to be essential for *M. tuberculosis* viability. The absolute requirement of Cyp121 might be due to a toxic effect of cyclic dityrosine or an essential function of mycocyclosin itself (Belin et al., 2009). Cyp121 has been shown to strongly bind to azoles, a class of antimycobacterial compounds, and might represent their main intracellular target (McLean et al., 2008). The structure of mycocyclosin necessitates that the two tyrosine side chains must be positioned on the same face of the DKP heterocycle during enzyme catalysis presumably facilitated by rotation around the C_α_–C_β_ bonds. In addition to positioning the two aromatic *meta* carbons in close proximity to one another, they must be activated to be able to form a C–C bond. This likely happens via a two-step radical mechanism that successively generates stabilized radicals in both aromatic rings that after positioning of both side chains combine to establish a new C–C bond.

The nocazine family of DKP-containing compounds is an example of natural combinatorial biosynthesis where a number of structurally related molecules is created through the action of a small number of promiscuous modification enzymes (Giessen et al., 2013a). Nocazines like the cytotoxic antibiotic neihumicin, have been isolated from different actinobacterial genera, including *Nocardiopsis, Streptomyces*, and *Micromonospora*. (Wu et al., 1988; Yang et al., 1988; Yokoi et al., 1988). By methylating the DKP carbonyl oxygen the *S*-adenosyl-L-methionoine (SAM)-dependent *O*-methyltransferase Ndas_1145 stabilizes the enole/imide resonance structure of one of the DKP amide bonds in a modified cyclo(L-Phe-L-Phe) substrate [**Figure 1**, reaction (10)]. This creates an extended planar conjugated system that might be crucial for its cytotoxicity. It is likely that Ndas_1145 is also able to methylate the DKP-ring nitrogens, as observed in the nocazines A/B/C, XR330, and XR333 (Giessen et al., 2013a).

**FIGURE 2:**
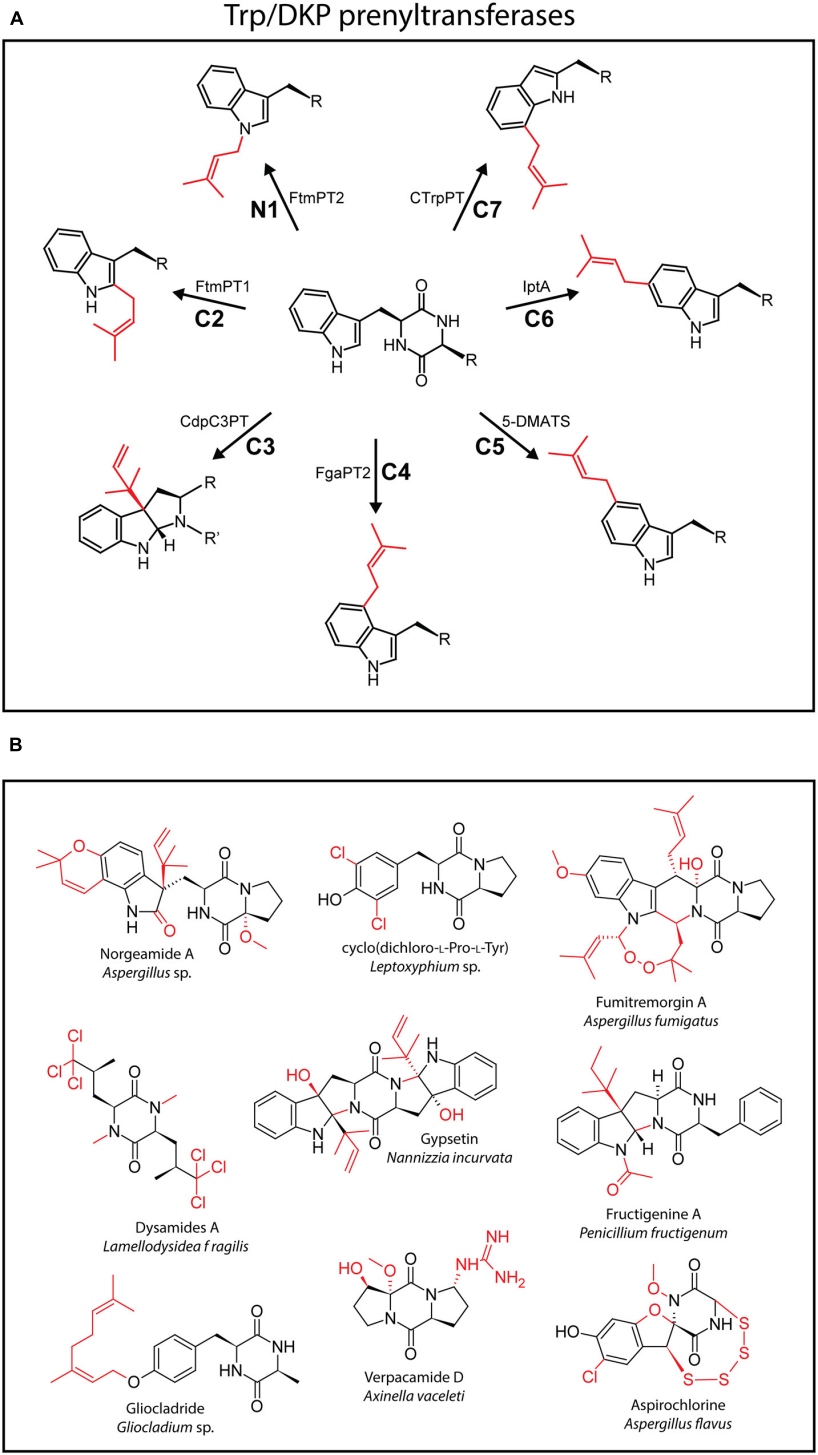
**(A)** Schematic representation of the (reverse) prenylation of all available positions in the indole ring of tryptophan catalyzed by different prenyltransferases (PTs). **(B)** Selection of structurally complex and unusual DKP-containing natural products. Unusual functionalities are highlighted in red.

Tryptophan-containing CDPs are among the most numerous DKP-containing natural products known (Borthwick, 2012). Especially fungi produce a bewildering array of structurally complex bioactive tryptophan-containing DKPs, including brevianamide F, norgeamide A, fumitremorgin A, and gypsetin among many others (Borthwick, 2012). What makes tryptophan an interesting scaffold for modification by tailoring enzymes is the rich nucleophilic chemistry of its indole ring where all positions are susceptible to electrophilic modification. Prenylation of the indole side chain using either isopentenyl pyrophosphate (IPP) or dimethylallyl pyrophosphate (DMAPP) as cofactors carried out by various types of prenyltransferases (PTs) may represent the most common modification found in tryptophan-containing CDPs. Over the last ten years, PTs that are able to modify all positions of the indole heterocycle in DKPs have been characterized and represent a valuable repertoire of diversification catalysts (**Figure 2A**) (Yu et al., 2012). Prenylations that expand the carbon skeleton of a natural product and may hugely influence its lipophilicity are very often crucial for their biological activity and ability to diffuse through cell membranes (Li, 2009a,b). The recent identification of a cyclo(L-Trp-L-Trp) producing CDPS in *Actinosynnema mirum*, a very small high-yielding biocatalyst, might further encourage the engineering of synthetic pathways for the production of various prenylated and further modified “unnatural” natural products which so far has relied on using very large NRPSs (*vide infra*; Giessen et al., 2013b).

Besides the various tailoring enzymes discussed above and described in the literature, many highly complex modified CDPs have been isolated where the responsible enzymes haven’t been identified or characterized so far. To highlight the hidden diversity of DKP-modifying enzymes a number of structurally intriguing natural products are shown in **Figure 2B**. Modifications found in those secondary metabolites include unusual oxidative cyclization reactions (norgeamides A; Grubbs et al., 2007), carbonyl (norgeamides A), oxoether (aspirochlorine; Klausmeyer et al., 2005), and peroxide (fumitremorgin A; Deveau et al., 2001) formations as well as hydroxylations (gypsetin etc.; Nuber et al., 1994), all likely introduced by oxidoreductase class enzymes. In addition, unusual transferase reactions that install geranyl (gliocladride; Yao et al., 2007), acetyl (fructigenine; Arai et al., 1989), and guanidinium (verpacamide D; Vergne et al., 2006) functionalities can be inferred from the structures shown. Finally, the structures of different chlorinated compounds [dysamides A, cyclo(dichloro-L-Pro-L-Tyr) and aspirochlorine] as well as a tetrasulfide-containing molecule (aspirochlorine) are shown (Klausmeyer et al., 2005). Halogenations have been reported in various natural products most often catalyzed by flavin- or α-ketoglutarate/Fe^II^-dependent halogenases, while the tetrasulfide functionality might arise through a similar GSH/PLP-dependent mechanism as the disulfide bridge of gliotoxin (Chankhamjon et al., 2014).

Given this possibly immense and so far unknown and untapped pool of biocatalysts that exists in microbial secondary metabolism, efforts directed toward the discovery and characterization of new small molecule tailoring enzymes have been intensifying over the past decade (Iqbal et al., 2012; Nguyen et al., 2012; Bologa et al., 2013). The ever-increasing advancements in DNA-sequencing and metagenomics, combined with ever-more sophisticated genome mining and computational discovery approaches focusing on previously underexplored environments and niches gives metabolic engineers and synthetic biologists the chance to reveal and harness the diversity of chemical transformations that has evolved over billions of years, thus shedding light on part of what has been referred to as microbial dark matter (Marcy et al., 2007; Rinke et al., 2013).

## Application of DKP Tailoring Enzymes in Combinatorial Biocatalysis

In this review, combinatorial biosynthesis is defined as the *in vivo* or *in vitro* combination of natural or engineered enzymes stemming from different pathways and/or organisms to generate either a specific high value target compound or a whole range of related structural variants for subsequent bioactivity screening. We differentiate this combinatorial biosynthesis approach where all of the chemical transformations are carried out by biocatalysts with chemoenzymatic synthesis, which combines the use of enzymes to carry out particularly challenging transformations with traditional organic chemistry.

In nature, many microbes rely on biosynthetic strategies that combine promiscuous enzymes from different biosynthetic pathways to either synthesize one functional product or to generate structural diversity. Examples include the assembly of the siderophores erythrochelin (Lazos et al., 2010; Robbel et al., 2010) and rhodochelin (Bosello et al., 2011) which relies on enzymes located in more than one distinct gene cluster and the generation of a family of pyrrolamide antibiotics that has recently been shown to rely on two separate genetic loci (Vingadassalon et al., 2015). Indeed, alternative modification enzymes outside of core biosynthetic gene clusters often form subclusters which are then able to join producing entirely new clusters (Fischbach et al., 2008). The retention of subclusters and newly joined hybrid clusters strongly depends on their usefulness for the producing organism. New non-functional gene clusters that don’t confer any evolutionary advantage to the host would have a limited evolutionary lifetime. These considerations underscore the importance of natural selection as a driving force of chemical innovation through diversification and subsequent selection over evolutionary time. This problem can be circumvented by the construction of artificial hybrid gene clusters and engineered organisms whose survival depends on the retention of the genes responsible for the production of a certain compound or set of compounds.

*In vivo* combinatorial biosynthesis can be accomplished through three distinct strategies. Firstly, using precursor-directed biosynthesis, where the promiscuity of biosynthetic enzymes is exploited to introduce non-native building blocks into a natural product scaffold. Secondly, employing enzyme-level modifications resulting in mutant enzymes with new functionalities and thirdly, pathway-level engineering where different enzymes are combined to generate completely artificial biosynthetic pathways (Sun et al., 2015).

**FIGURE 3:**
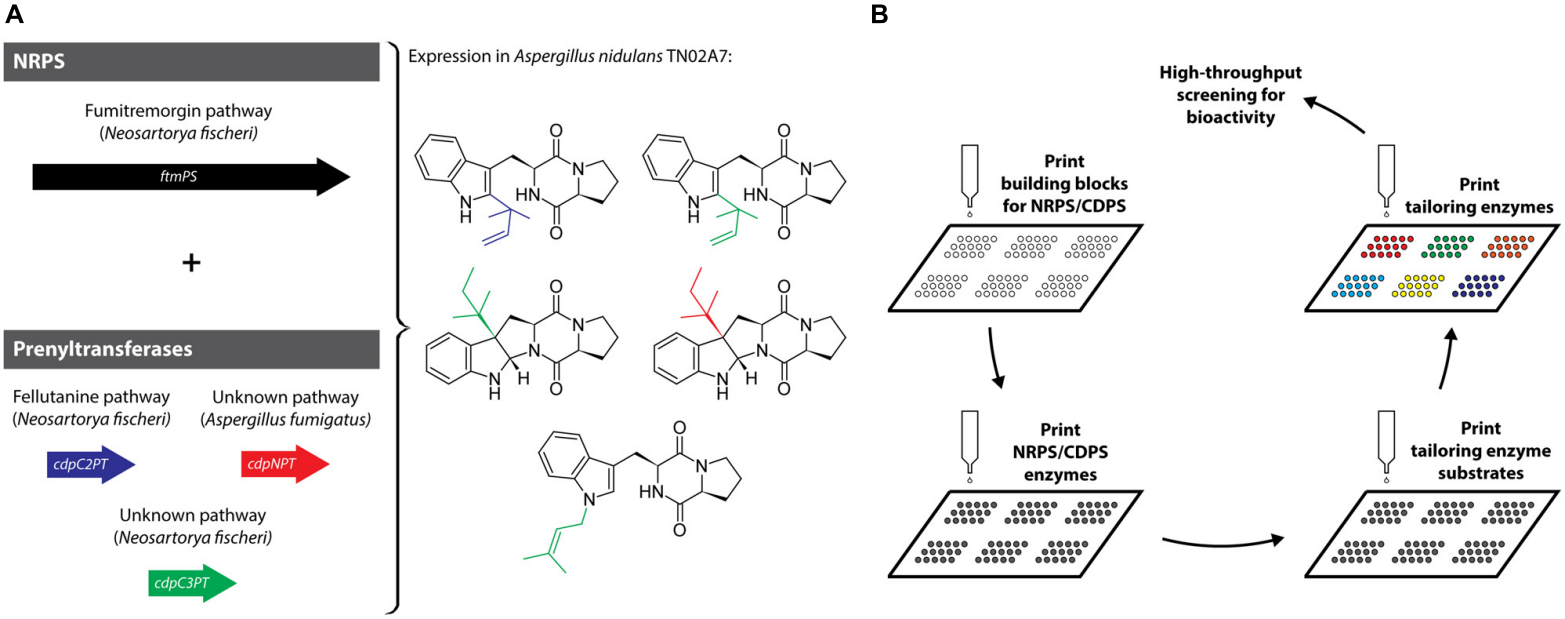
**(A)** *In vivo* combinatorial biosynthesis employing a dimodular NRPS and three PTs resulting in the generation of a set of differently (reverse) prenylated DKPs. **(B)** *In vitro* combinatorial biosynthesis approach based on printing biosynthetic enzymes and substrates on microarray-chips coupled to high-throughput screening.

In a recent example of *in vivo* pathway-level combinatorial biosynthesis, a dimodular NRPS that generates cyclo(L-Trp-L-Pro), was separately combined with three different PTs in *A. nidulans* resulting in differently prenylated DKP products (**Figure 3A**) (Wunsch et al., 2015). The authors used enzymes from four different pathways and two different organisms to predictably introduce prenylations and reverse prenylations to a DKP substrate. Modified CDPs based on the cyclo(L-Trp-L-Pro) scaffold represent the most abundant and structurally diverse family of DKP-containing natural products. This might be because proline adopts a *cis*-conformation about the Xaa-Pro amide bond making it prone to DKP-formation (Borthwick, 2012). By focusing on this DKP-family the authors open the door for the generation of a large number of structurally diverse natural products and their analogs with potentially new and improved biological activities.

Diversity-oriented combinatorial biosynthesis *in vitro* represents an alternative approach where purified biosynthetic enzymes and their substrates are successively printed onto a microarray-chip (**Figure 3B**) (Kwon et al., 2012). Pathway reconstruction or diversification *in vitro* can be used to characterize individual enzymes and identify pathway intermediates. Although enzymes need to be purified and substrates provided separately, this approach has some interesting advantages compared with *in vivo* strategies. *In vitro* diversification is not restricted by intermediate or product toxicity, limited availability of intracellular precursors or unanticipated regulatory mechanisms. Maybe most importantly, chip-based *in vitro* biosynthesis can be easily integrated with high-throughput screening strategies (Kwon et al., 2007). Generally, natural product classes that rely on the action of one key enzyme followed by scaffold diversification by modification enzymes are best suited for this approach, including polyketides, non-ribosomal peptides, including DKP-containing compounds, and terpenoids.

Although great advances have been made in combinatorial biosynthesis over the last years with new whole genome sequencing methods revealing an ever increasing number of biocatalysts for the synthetic biologist to choose from, an increase in understanding biosynthetic logic at the enzyme, pathway, and organism level and advancements in enzyme engineering, many challenges still remain (Sun et al., 2015). Often, biosynthesis approaches suffer from low yields which prevents their use in a commercial setting. This challenge could be tackled through concerted enzyme and metabolic engineering efforts and by finding the optimal expression host (Pickens et al., 2011). Chassis optimization may be necessary in cases where a gene product turns out to be toxic to the producing organism or when endogenous regulatory mechanisms lower the yield of the engineered pathway (Fisher et al., 2014). Although DKPs are generally membrane-permeable and accumulate in the growth medium, certain modifications may prevent them from crossing the cell membrane which would make their isolation more difficult. In approaches aimed at generating large compound libraries for drug discovery efforts, a large number of combinatorially generated biocatalyst combinations must be generated in a high-throughput fashion. This was traditionally limited by conventional cloning approaches, but could be circumvented by using new and rapid DNA synthesis and assembly techniques (Chao et al., 2014; Cobb et al., 2014). Finally, integrating combinatorial biosynthesis and the resulting large compound libraries with rapid high-throughput screening methods is of paramount importance. Those efforts could be guided by combining computational with structural and bioactivity analyses (Winter et al., 2011; Damborsky and Brezovsky, 2014).

As outlined above, combinatorial biosynthesis has come a long way and is now at the verge of being widely and easily applicable in drug discovery, medicinal chemistry, and synthetic biology. This is especially true for DKP-containing compounds that have proven their value as important molecular scaffolds in various fields in the past and will become even more useful with the increasing application of tailoring enzymes in metabolic engineering and synthetic biology approaches in the future.

## Conflict of Interest Statement

The authors declare that the research was conducted in the absence of any commercial or financial relationships that could be construed as a potential conflict of interest.
